# Can laparoscopic nerve-sparing ultra-radical hysterectomy play a role in locally advanced cervical cancer? A single-center retrospective study

**DOI:** 10.3389/fonc.2022.1003951

**Published:** 2022-10-25

**Authors:** Wei-wei Wei, Hong Zheng, Panqiu Shao, Xia Chen, Yi-fei Min, Bin Tang, Hui-ting Sun, Ji-ming Chen, Ru-xia Shi

**Affiliations:** ^1^ Department of Gynecology, The Affiliated Changzhou No. 2 People’s Hospital of Nanjing Medical University, Changzhou, China; ^2^ Department of Reproductive Center, The Affiliated Changzhou No. 2 People’s Hospital of Nanjing Medical University, Changzhou, China

**Keywords:** locally advanced cervical carcinoma, nerve-sparing radical hysterectomy, laparoscopic surgery, concurrent chemoradiotherapy, disease-free survival

## Abstract

**Background and objectives:**

The objective of this study is to investigate the outcomes of concurrent platinum-based chemoradiation therapy (CCRT), laparoscopic nerve-sparing ultra-radical hysterectomy (LNSURH), and open radical hysterectomy (ORH) on patients with locally advanced cervical carcinoma (LACC).

**Methods:**

A single-center retrospective study was conducted on LACC patients who received CCRT, ORH, or LNSURH from January 2011 to December 2019. Data on age, tumor size, overall survival (OS), disease-free survival (DFS), and early and late morbidities were collected. After 24 months of treatment, patients were asked a series of questions about their urinary, bowel, and sexual activities. Early morbidities were defined as those occurring during or within a month of treatment, whereas late morbidities and complications were defined as those occurring a month after treatment. The postoperative complications were classified with reference to the Clavien–Dindo classification (CD) system.

**Results:**

The Kaplan–Meier curves revealed no significant differences in OS and DFS among the three groups (*P* = 0.106 for DFS and *P* = 0.190 for OS). The rates of early complications in the CCRT group were comparable with those in the operated groups (*P* = 0.46). However, late complications were significantly lower in the ORH and LNSURH groups relative to those in the CCRT group. The scores of urinary and bowel functions were restored to the pretreatment state, although the sexual function scores were not satisfactory.

**Conclusions:**

The treatments of CCRT, ORH, and LNSURH can be considered options for patients with LACC, as their OS and DFS showed no significant difference. In addition, LNSURH exhibited a lower incidence of late complications and high sexual function scores.

## Introduction

Cervical cancer is the fourth most common malignancy in women across the world (1). Radical hysterectomy (RH) represents the classical treatment for early-stage cervical cancer. Locally advanced cervical carcinoma (LACC) is larger with cervical carcinoma > 4 cm and stage IB2 or IIA2 (2). The exploration of the treatment for LACC has never stopped. According to the National Comprehensive Cancer Network (NCCN) guidelines, both RH and CCRT can be applied as the treatment approach for LACC. However, the NCCN guidelines published in 2014 clearly stated that, for the treatment of stage IB2 (> 4 cm) and IIA2 (> 4 cm) LACC, concurrent chemoradiotherapy should be preferred over surgery. However, due to the differences in the radiotherapy levels and resources, as well as based on the patient’s willingness to undertake surgical treatment, surgical treatment remains an indispensable part of the treatment regimen for LACC.

CCRT is the first-line treatment option for LACC (3). However, LACC patients routinely treated with CCRT have demonstrated a poor prognosis, with about one-third of the patients relapsing within 18 months of CCRT (4), with a 5-year survival rate of 50–60% (5). In developing countries such as China, patients often present with different stages of LACC (6). In stages IB3 and IIA2, the possible causes of relapse have been reported to be larger tumors and residual tumor tissues after CCRT. Blidaru et al. (7) reported that, in 2019, 30–40% of patients with surgery for LACC who were following CCRT had residual tumor tissues on pathology examination of their hysterectomized specimen. Despite LACC being larger and with a high possibility of positive lymph nodes, positive parametria, or positive surgical margins that augment the risk of recurrence and the rate of adjuvant radiation after surgery, RH is a treatment option for locally advanced tumors.

The *New England Journal of Medicine* reported that, in 2018, minimally invasive RH was associated with a poor prognosis relative to that with open RH among women with early-stage cervical cancer (8). However, this conclusion has been questioned by several scholars, and the surgical method has been improved; as a result, minimally invasive RH has been deemed a safe approach in terms of the oncological outcomes (9, 10). In 2015, we reported the surgical procedure of LNSRH, with a disease-free survival rate of 90.6% in the LNSRH (11). Based on the results of this past study, we continued to conduct laparoscopic nerve-sparing ultra-radical hysterectomy (LNSURH), open radical hysterectomy (ORH), and CCRT, after providing the patients the relevant information.

To investigate whether patients with LACC can benefit from LNSURH, we evaluated the outcome of LNSURH, RH (RH), and CCRT in patients with LACC. The disease-free survival (DFS), OS, and complications were recorded and analyzed to determine the prognosis and quality of life of these patients.

## Materials and methods

### Study design and patient selection

LACC patients in stages IB3 and IIA2 (74) who had received ORH (29), LNSURH (20), or CCRT (25) from the Affiliated Changzhou No. 2 People’s Hospital of Nanjing Medical University between January 2011 and December 2019 were enrolled in this study. All surgical cases were treated by the same surgical team (Prof. Ru-Xia Shi et al.). This study was approved by the hospital’s ethics committee (approval number: [2019] YLJSA011).

The subject inclusion criteria were as follows: (1) patients at stages IB3 or IIA2, as defined by the International Federation of Gynecology and Obstetrics (FIGO) staging system (2018) before treatment and with pathologically confirmed cervical squamous cell carcinoma, adenocarcinoma, or adenosquamous; and (2) patients who had RH, LNSURH with pelvic and para-aortic lymphadenectomy, or CCRT.

The subject exclusion criteria were as follows: (1) combination with other malignant tumors, (2) incomplete medical records, and (3) abnormal vital organ functions.

### Surgical procedure

The surgical procedure for LNSURH with pelvic and para-aortic lymphadenectomy was performed under general anesthesia in the dorsolithotomy position. The operation platform was performed by a five-port laparoscopy. The para-aortic lymph node dissection was performed routinely up to the inferior mesenteric artery emergence. If the intraoperative frozen section examination indicated a positive common iliac lymph node, it was performed up to the renal vein level.

A laparoscopic pelvic lymphadenectomy was performed following para-aortic lymphadenectomy. The critical steps of nerve preservation were the development of the anatomical space, identification, selective transection of the uterine nerve branches (UNBs) of the inferior hypogastric plexus (IHP), and sparing of the vesical nerve branches (VNBs). As cervical carcinoma patients at stage IB3 or IIA2 with larger tumor and parametrial infiltration were not deemed suitable for the nerve-sparing procedure, contralateral or partial nerve-sparing RH was performed to preserve some part of the IHP and the pelvic splanchnic nerves (12).

**Figure 1:**
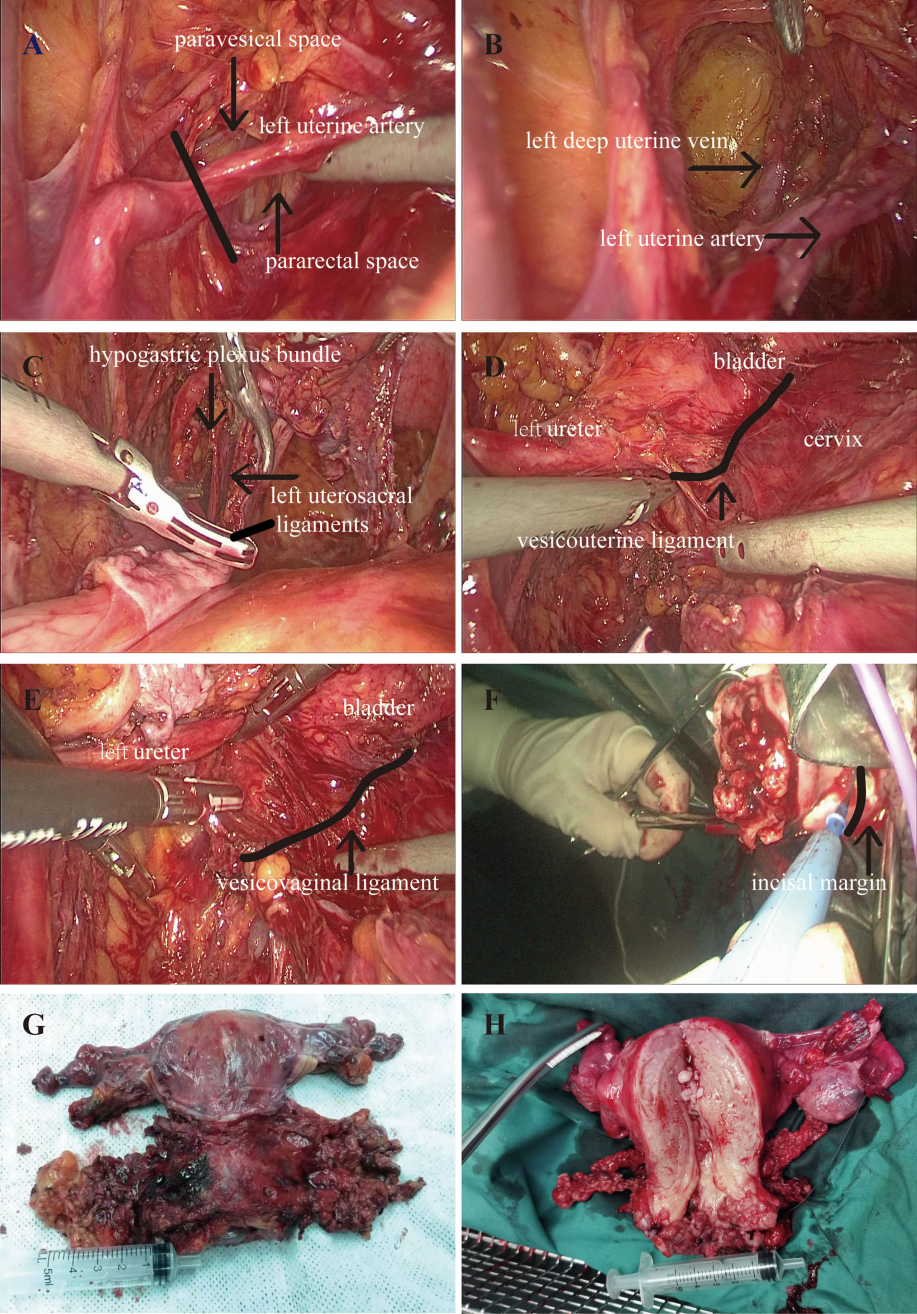
Perioperative picture. **(A)** Resection of the uterine artery. **(B)** Isolation of the deep uterine vein. **(C)** Resection of the uterosacral ligaments. **(D)** Resection of the vesicouterine ligament. **(E)** Resection of the vesicovaginal ligament. **(F)** The removal of the uterus. **(G, H)** Ultra-radical hysterectomy specimen.

LNSURH has a wider parametrial excision and safeguards the pelvic splanchnic nerve from long-term postoperative complications (e.g., urinary dysfunction, sexual dysfunction, and bowel motility disorders). The key point of LNSURH is depicted in **Figure 1**. The procedure is summarized as follows:

Step 1: Resection of the uterine artery at the starting position of the internal iliac artery following the development of the pararectal space and paravesical space (**Figure 1A**).

Step 2: Continue downward to separate the pararectal and paravesical spaces, followed by exposure and isolation of the deep uterine vein. We then exposed the deep uterine vein to reveal the pelvic splanchnic nerve beneath it. Next, we closed the uterine bilateral arteries and veins and then excised the parametrial tissues so as to reduce blood loss (**Figure 1B**).

Step 3: We pushed the hypogastric plexus bundle laterally and resected the root of the distal uterosacral ligaments (**Figure 1C**).

Step 4: Vesicouterine ligament (VUL) is located between the bladder and the cervix and is a lamellar structure (**Figure 1D**). The vesicovaginal ligament (VVL) is located between the bladder and the vagina at the level of the vaginal fornix, which is a posterior portion of the VUL. The VVL is exposed after the excision of the VUL (**Figure 1E**). Vesical vein is then transected at the edge of the bladder, disconnecting the bladder from the cervix and the upper vagina. The excision of the VUL and the VVL was performed close to the bladder with a wider parametrial excision.

Step 5: We next conducted cross-shaped IHP and isolated the uterine branch from the plexus. The uterine branch division was performed to create a T-shaped nerve plane of the IHP. The T-shaped nerve plane was pushed down before the radical excision of the paracolpium to avoid nerve damage.

Step 6: The uterus was removed through the vagina, and the length of the vagina was 3 cm (**Figure 1F**).

Step 7: The ultra-radical hysterectomy specimen was obtained (**Figures 1G, H**).

### Data collection

The data relating to the age of the patients, the tumor size, early complications, and late complications were collected. All patients diagnosed with cervical cancer routinely underwent medical imaging to evaluate the abdomen and the pelvis, respectively, before treatment. LACC was diagnosed by two deputy chiefs or experts supervising the gynecological oncologists. These patients with LACC then underwent RH and LNSURH with pelvic and para-aortic lymphadenectomy or CCRT.

The patients in the RH and LNSURH groups were given adjuvant radiotherapy in case of a risk of tumor recurrence postoperatively. The risk factors included (1) 1 of the high-risk factors such as lymph node metastases, positive resection margins, and parametrial invasion, or (2) >1 of the other risk factors that include deep stromal invasion and lymphovascular space invasion (LVSI) (13–16).

The early complications included myelosuppression, hypohepatia, radiation enteritis, radiocystitis, pelvic lymphatic cyst, angiolymphitis, ureteral vaginal fistula, and radiothermitis. Late complications included obstructive nephropathy, lymphatic reflux disorder, and colorectal fistula. The ureteral vaginal fistula was treated with ureteral stenting. All early complications improved after the treatment.

The Clavien–Dindo (CD) classification system was applied to analyze the post-operative complications (17). It was defined as lower than or equal to grade II (not requiring surgical, endoscopic, or radiological intervention) and higher than or equal to grade III (requiring surgical, endoscopic, or radiological intervention or life-threatening complication or death of a patient).

After discharge from the hospital, the patients were followed up *via* a telephonic and outpatient care interview conducted every 3 months. The follow-up data included the duration of the follow-up; the general health status; complications; time of cancer recurrence; urinary, bowel, and sexual functions; and mortality.

During the follow-up, at least 24 months after the operation or CCRT, the patients were asked to answer a series of questions about their urinary, bowel, and sexual functions. The self-assessed questionnaires consisted of five questions on sexual satisfaction, dyspareunia, defecation condition, urinary incontinence, and urination requiring abdominal assistance, according to the article published by Zhuoyu Sun in 2020 (18). The scores for each question ranged from 0 to 3, with higher scores indicating a better quality of life.

DFS was considered as the period from surgery or CCRT to cancer recurrence, as identified by biopsy or evaluation by medical imaging. In case of no cancer recurrence, the last follow-up examination or death was considered as the DFS. Overall survival (OS) was regarded as the period from the time of treatment including surgery and CCRT until death from cervical cancer.

### Statistical analysis

We analyzed the factors associated with OS and DFS by multivariate logistic regression analyses. Comparison of continuous data was performed by one-way analysis of variance (ANOVA), and continuous data were expressed as the mean ± *SD*. Comparison of the categorical data was performed by the chi-square test, and categorical data were expressed as percentages. DFS and OS were detected by Kaplan–Meier analysis. We calculated *P* values by log-rank test. *P* < 0.05 was considered to indicate statistical significance. All data were analyzed by SPSS 20.0 (SPSS, IBM, New York, NY).

## Results

### Participant characteristic comparisons

In our study, we assessed 74 patients, with 25, 29, and 20 patients assigned to the CCRT, RH, and LNSRH groups, respectively. Statistical analysis revealed that there were no statistically significant differences in the BMI and tumor size. However, when compared with the CCRT group, the LNSURH and RH groups were significantly younger (**Table 1**).

**Table 1:** Characteristics of the study participants.

Characteristics	CCRT group	ORH group	LNSURH group	*P*
	(*N* = 25)	(*N* = 29)	(*N* = 20)	
Age, years (mean ±*SD*)	55.2 ±11.7	51.7 ±8.2	47.1 ±10.5	0.03
BMI	22.69 (3.28)	23.69 (3.08)	23.14 (2.66)	0.48
Tumor size, cm (mean ±*SD*)	5.00 (0.94)	4.53 (0.57)	4.80 (0.71)	0.1
FIGO stage, *N* (%)				0.04
IB3	15 (60.00)	18 (62.07)	11 (55.00)	
IIA2	10 (40.00)	11 (37.93)	9 (45.00)	
Pathology, *N* (%)				0.141
Squamous cell carcinoma	21 (84.00)	27 (93.10)	15 (75.00)	
Adeno/adenosquamous carcinoma	4 (16.00)	2 (6.89)	5 (25.00)	

### Comparisons of survival after the therapy survival among the three groups

A total of 74 patients showed a median postoperative follow-up time of 39.6 months (0–115 months). The DFS rates were 84.0, 75.9, and 90.0%, whereas the OS rates were 96.0, 93.1, and 100% in the CCRT, ORH, and LNSURH groups in 3 years, respectively. The median DFS times were 49, 53, and 35 months, and the median OS times were 48, 50, and 28 months in the CCRT, ORH, and LNSURH groups, respectively.

**Figure 2:**
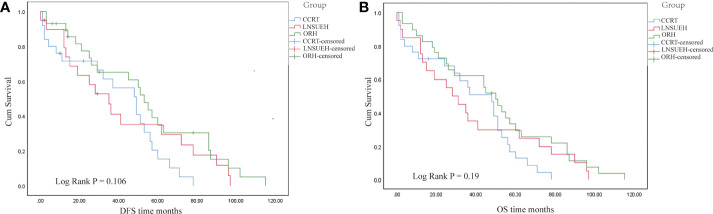
Kaplan–Meier survival analyses. **(A)** Comparisons of the disease-free survival (DFS) periods among the three study groups. **(B)** Comparisons of the overall survival (OS) periods among the three groups.

The Kaplan–Meier analysis showed that there was no significant difference in the OS and DFS among the CCRT, ORH, and LNSURH groups (*P* = 0.106 for DFS and 0.190 for OS, **Figures 2A, B**, respectively).

### Early and late complications

The rate of early complications was not statistically significantly different among the CCRT, ORH, and LNSURH groups (*P* = 0.46). When compared with the CCRT group, the rate of late complications in the ORH and LNSURH groups was markedly lower (**Table 2**).

**Table 2:** Rate of complications in the CCRT, ORH, and LNSURH groups.

Complications	CCRT group	ORH group	LNSURH group	*P*
	(*N* = 25)	(*N* = 29)	(*N* = 20)	
Early complications *N* (%)	17 (68)	18 (62.1)	10 (50)	0.463
Myelosuppression	12	10	6	
Hypohepatia	4	4	3	
Radiation enteritis	6	4	2	
Radiocystitis	3	1	5	
Pelvic lymphatic cyst	0	2	1	
Angiolymphitis	0	1	1	
Ureteral vaginal fistula	0	1	1	
Radiothermitis	1	0	0	
Late complications	5 (20)	0	1 (5)	0.015
Obstructive nephropathy	2	0	1	
Lymphatic reflux disorder	1	0	0	
Colorectal fistula	2	0	0	0.352
CD				
Grade ≤ II	19	17	10	
Grade≥ III	3	1	1	

The early complications included myelosuppression, hypohepatia, radiation enteritis, radiocystitis, pelvic lymphatic cyst, angiolymphitis, ureteral vaginal fistula, and radiothermitis. The same patient often exhibited several early complications simultaneously; hence, the times of early complications were counted by the patients experiencing early complications.

CD, Clavien–Dindo classification.

### Post-treatment functional evaluation

During follow-up, at least 24 months after the procedure, the patients were asked to answer a series of questions about their urination, defecation, and sexual functions. A total of eight patients from the 25 patients in the CCRT group, six of the 29 patients in the ORH group, and nine of the 20 patients in the LNSURH group answered the questionnaires.

The scores of sexual functions were 0.25 ± 0.66, 0.83 ± 1.21, and 1.56 ± 2.31 in the CCRT, ORH, and LNSURH groups, respectively. The scores of urinary and bowel functions were restored to the pretreatment state in all three groups, albeit the sexual function was not satisfactory (**Table 3**).

**Table 3:** The scores for each question in the CCRT, ORH, and LNSURH groups.

Scores (mean ±*SD*)	CCRT group	ORH group	LNSURH group
	(*N* = 25)	(*N* = 29)	(*N* = 20)
Sexual function	0.25 ± 0.66	0.83 ± 1.21	1.56 ± 2.31
Rectum function	2.63 ± 0.99	3.00 ± 0.00	2.89 ± 0.31
Bladder function	5.88 ± 0.33	5.67 ± 0.75	5.56 ± 0.68
Aggregate score	8.50 ± 1.00	9.50 ± 1.61	10.00 ± 2.75

## Discussion

Cervical cancer is one of the most common gynecological malignancies across the world. Based on the latest statistics, more than 311,000 women worldwide died of cervical cancer in 2018 alone (19, 20). Most of these patients were diagnosed in the late stage of the disease, making timely estimation of the clinical grade the most important prognostic factor of this tumor. The treatment of LACC has always been a hot issue worthy of research and discussion. Clinically, the main treatment approach for LACC includes radiotherapy and chemotherapy, although the treatment outcome is generally poor. This type of tumor is not easy to control locally, which makes it difficult to operate and easy to relapse and metastasize even after the operation, and the 5-year survival rate is low (21, 22). The latest guideline from the NCCN recommends CCRT, including external radiotherapy and brachytherapy, which is the standard treatment approach for LACC patients (23, 24). Cochrane meta-analysis completed by GOG and the Radiation Therapy Oncology Group (RTOG) demonstrated that, for women with LACC, the 5-year survival rate of CCRT was increased by 6% when compared with that by radiotherapy alone (hazard ratio [HR] = 0.81, *P* < 0.001) (25, 26). Li et al. (27) reported that the 5-year overall response rate of CCRT for the treatment of LACC patients was 67%. A retrospective study compared the curative effects of paclitaxel/ifosfamide/platinum (TIP) and paclitaxel/platinum (TP) on patients with metastatic, recurrent, or persistent cervical cancer. They found that TIP exhibited a higher remission rate than TP without increasing the risks of severe complications (28). Kalaghchi et al. (29) reported that LACC patients exhibited good tolerance to cisplatin and paclitaxel combined chemotherapy and radiotherapy, albeit the tumor response and PFS did not show any improvement. When compared with CCRT combined with platinum monotherapy, CCRT combined with platinum monotherapy could improve the OS and PFS of patients with LACC, albeit it also increased the adverse reactions caused by several chemotherapeutic drugs. A systematic review with meta-analysis that evaluated the efficacy of CCRT and neoadjuvant chemotherapy followed by radical surgery (NACT+S) revealed that, when compared with the CCRT group, the incidence of diarrhea, rectal, and bladder complications in the NACT+S group was lower, although NACT+S exhibited no survival advantage for patients with IB2-IIB cervical cancer (30). Until now, there exists no consensus on whether NACT can significantly improve the prognosis of cervical cancer (31). Therefore, in clinical practice, it is extremely important to select the appropriate chemotherapy scheme in accordance with the patient’s actual tolerance. Therefore, the choice of LACC treatment remains a huge problem in the currently available treatment ^-^modalities (32).

However, owing to the difference in the advancement of medical and healthcare facilities across the world, there is a deviation in clinical staging before surgery, an imbalance in the radiotherapy resources, limitations of regional-related medical conditions, and the subjective choice made by patients, considering that a considerable number of patients continue to opt for surgical resection as an initial treatment (33–35). In recent years, with the development of the minimally invasive concept, surgical instruments, and relevant technology, laparoscopic RH for cervical cancer has been proved to be safe and effective, thereby gradually replacing the traditional open surgery approach (36, 37). However, the radical effect of this operation and the suitability of its application scope remain controversial. Recent research reports have raised serious concerns about the oncological safety of endoscopic surgery for cervical cancer and highlight the remarkable and alarming increase in the recurrence rate (38, 39). With the rising trend of minimally invasive surgery and the development of laparoscopic technology, along with the advantage of a clear vision offered by the latest laparoscopy techniques, it has become more conducive to preserving the pelvic autonomic nerve structure and further improving the quality of surgery. Since 2008, our research group has been exploring the precise anatomy of radical operation of cervical cancer and the improved operation method for laparoscopic nerve-sparing RH (LNSRH), thereby mastering solid surgical skills and accumulating significant case data. Our previous research preliminarily confirmed that LNSRH can preserve the urinary, colorectal, and sexual functions and arrest lymph node metastasis, rather than the type of hysterectomy, which is independently related to the DFS and OS (40). Several research reports across the world have supported and validated the advantages of nerve-sparing minimally invasive RH (NS-MRH) operation in improving bladder functions and the safety of reducing the pelvic floor dysfunction rate (41, 42). In 2016, a multicenter prospective cohort study on 76 patients with IB2 and IIA2 cervical cancer whose local tumors were >6 cm in size completed laparoscopic nerve-sparing RH (LNRH) and laparoscopic RH (LRH) after neoadjuvant chemotherapy. Their results asserted that LNRH is safe and feasible in the treatment of LACC, although the study followed up the patients for only 1 year and did not conduct any prognosis evaluation (43). A retrospective study evaluated the survival outcome of minimally invasive radical surgery (MI-RS) versus open radical surgery (O-RS) in LACC patients managed by surgery after CT/RT through propensity score analyses. MI-RS and O-RS were found to be associated with similar rates of recurrence, and there was no difference in the early or late complications (44). Moreover, the feasibility of secondary radical resection positively impacts the survival of recurrent LACC patients submitted to multimodality primary treatments, thus prompting practitioners treating patients with recurrence from cervical cancer to consider a second surgery in the armamentarium of potential therapies (45).

All surgery candidates in this study preferred adjuvant radiotherapy and chemotherapy according to the high-risk factors suggested by their postoperative pathological outcomes. After a median of 39.6 months’ long-term follow-up, our results showed that, when compared with conventional chemoradiotherapy, LNSURH plus chemoradiotherapy had no significant difference in the OS and DFS in LACC patients, suggesting that LNSURH preserved the pelvic nerve but did not increase the postoperative recurrence rate. It is therefore recommended that the postoperative survival rate of patients is related to the scope of surgery and the implementation of the principle of no tumor, albeit it has no obvious correlation with the surgical method. After the pelvic nerve is preserved, the bladder function of the patient recovers quickly. Moreover, this way, patients with risk factors who need postoperative adjuvant radiotherapy can receive treatment at the earliest. In this study, the early complications of CCRT, ORH, and LNSURH patients included myelosuppression, hypohepatia, and radiation enteritis, albeit the corresponding incidences were not statistically significantly different among the groups. The long-term follow-up of late complications demonstrated that the rectal and bladder functions recovered to the preoperative state and that the quality of life was improved, although the recovery of sexual functions was not satisfactory; in fact, it was lower than those reported previously (36), which may be attributed to the Chinese women’s sexual psychological worries. The results of the present study implied that active adjuvant therapy can help improve the prognosis of patients with LACC. Moreover, laparoscopic para-aortic and pelvic lymphadenectomy provides accurate information about the lymph node status and allows the development of individualized treatment plans for LACC patients, thereby avoiding false-negative (FN) and false-positive (FP) imaging results (46).

To analyze the reasons for good OS and DFS after LNSRH operation, the following factors should be considered: (1) based on nerve preservation, the operation scope is sufficient; and (2) strict implementation of the principle of being tumor-free: (A) removal of the uterus through the vagina; (B) flushing with plenty of water after the operation; (C) lymph node removal on time by bagging; and (D) ensuring that pneumoperitoneum is stable and instrument replacement is minimized.

Comparatively speaking, this retrospective small-sample study involved a selective deviation. Patients who selected LNSRH were younger than those in the CCRT and ORH groups, and they are more enthusiastic about undertaking laparoscopic surgery with nerve preservation. In addition, the quality of life was self-reported by the patients themselves, which implies the possibility of deviation in self-reporting. Therefore, for validation of the present findings, larger randomized trials and longer follow-ups are warranted.

## Conclusions

In summary, the present research supports that the prognosis of LACC patients should be determined by the scope of surgery and tumor-free outcome and not by the difference in the surgical approaches. No significant difference was noted in the OS and DFS among the three study groups, albeit there were more long-term complications of CCRT, such as vaginal fistula, ureteral obstruction (related to the uncleared primary lesion), and obstructive nephropathy. The response to the questionnaires revealed that the sexual life score of the LNSRH group was higher than that of the other two groups.

## Data availability statement

The original contributions presented in the study are included in the article/supplementary material. Further inquiries can be directed to the corresponding authors.

## Ethics statement

This study was approved by the ethics committee of Nanjing Medical University Affiliated Changzhou Second People’s Hospital (approval number: [2019] YLJSA011). The patients/participants provided their written informed consent to participate in this study. Written informed consent was obtained from the individual(s) for the publication of any potentially identifiable images or data included in this article.

## Author contributions

JC and RS designed the study and approved the manuscript. HZ and BT collected the clinical data. WW and HS prepared and wrote the original draft, with the reviewing of YM and XC. PS provided statistical methods. All authors contributed to the article and approved the submitted version.

## Funding

This work was supported by grants from the Changzhou Science and Technology Program (QN201931) and the Changzhou Sci and Tech Program (grant no. CJ20220077 and CJ20210110).

## Acknowledgments

The authors would like to thank those female patients who participated in the study and shared their experiences with us.

## Conflict of interest

The authors declare that the research was conducted in the absence of any commercial or financial relationships that could be construed as a potential conflict of interest.

## Publisher’s note

All claims expressed in this article are solely those of the authors and do not necessarily represent those of their affiliated organizations, or those of the publisher, the editors and the reviewers. Any product that may be evaluated in this article, or claim that may be made by its manufacturer, is not guaranteed or endorsed by the publisher.
